# Effects of Micro-Braiding and Co-Wrapping Techniques on Characteristics of Flax/Polypropylene-Based Hybrid Yarn: A Comparative Study

**DOI:** 10.3390/polym12112559

**Published:** 2020-10-31

**Authors:** Wenqian Zhai, Peng Wang, Xavier Legrand, Damien Soulat, Manuela Ferreira

**Affiliations:** 1 University of Lille, Ensait, Gemtex, F-59000 Roubaix, France; wenqian.zhai@ensait.fr (W.Z.); xavier.legrand@ensait.fr (X.L.); damien.soulat@ensait.fr (D.S.); manuela.ferreira@ensait.fr (M.F.); 2 University of Haute-Alsace, Ensisa, Lpmt, F-68000 Mulhouse, France; 3 University of Strasbourg, 67081 Strasbourg, France

**Keywords:** textile composites, natural fibers, micro-braiding, hybrid yarns, mechanical behavior

## Abstract

Micro-braiding and co-wrapping techniques have been developed over a few decades and have made important contributions to biocomposites development. In this present study, a set of flax/polypropylene (PP) micro-braided and co-wrapped yarns was developed by varying different PP parameters (PP braiding angles and PP wrapping turns, respectively) to get different flax/PP mass ratios. The effects on textile and mechanical characteristics were studied thoroughly at the yarn scale, both dry- and thermo-state tensile tests were carried out, and tensile properties were compared before and after the braiding process to study the braidabilities. It was observed that PP braiding angles of micro-braided yarn influenced the frictional damage on surface treatment agent of flax roving, the cohesive effect between PP filaments/flax roving, and the PP cover factor; PP wrapping turns of co-wrapped yarn had a strong impact on the flax roving damage and the PP coverage, which further influenced the characteristics. Micro-braided yarn and co-wrapped yarn with the same flax/PP mass ratio were compared to evaluate the two different hybrid yarn production techniques; it was proven that micro-braided yarn presented better performance.

## 1. Introduction

Biocomposites of thermoplastic polymers reinforced with natural fibers have received ever-increasing attention over the last few decades due to public awareness about global pollution and limited resources, being used in certain specific industrial areas such as the automotive, marine, and construction sectors [1,2,3]. So-called thermoplastic biocomposites possess a lower environmental impact but still meet desired characterization standards, in consideration of (1) mechanical properties of natural fibers, being strong enough over the other synthetic fibers as an alternative; (2) the reinforcing fiber orientation inside a polymer matrix, maintaining maximum parallel alignment towards the testing direction to obtain the best mechanical properties; and (3) poor interface adhesion and impregnation between natural fiber and thermoplastic polymers, since natural fibers are hydrophilic while polymer matrixes are typically hydrophobic. In addition, due to the high viscosities of thermoplastic polymer, impregnation of the thermoplastic matrix into continuous natural fibers is difficult [4,5].

A large number of studies on this issue consider hybrid yarns [6], which contain both the matrix and reinforcing components in their structure, enable the impregnation and shape forming process at the same time, and improve the thermoplastic resin distribution by decreasing effective flow distance, allowing good impregnation and mechanical performance of composites to be realized [7,8]. Hybrid yarns can be manufactured by various means; Alagirusamy et al. reviewed the different techniques of hybrid yarn manufacturing and explained their potency as thermoplastic composite medium materials [9]. Many studies applied hybrid yarn techniques with natural fibers for thermoplastic biocomposites, including micro-braiding, co-wrapping, and DREF. Khondker et al. produced jute/PLA and jute/ PP micro-braided yarns and conducted tensile and three-point bending tests to investigate the effects of molding condition on their mechanical and interfacial behavior [10]. Kobayashi et al. used micro-braiding as well to improve matrix impregnation of natural yarns. Hemp/PLA micro-braided yarns were created; their tensile and shear properties were assessed and showed good values [11]. Zhang et al. designed flax/PP wrap-spun hybrid yarn for biocomposites, in which PP filament was sparsely wrapped around a core of flax; it was observed that a composite based on twistless wrap-spun yarn provided better mechanical properties when compared to a composite reinforced with twisted yarn [12]. Similarly, Baghaei et al. designed a hemp/PLA wrap-spun hybrid yarn for biocomposites, in which PLA filament was sparsely wrapped around the mixed core of PLA fibers and hemp fibers; it was observed that the aligned hemp/PLA yarn composite possessed exceptional mechanical properties, including tensile, flexural, and impact strengths, and lower porosity and water absorption [13]. However, the sparsely wrapped yarns are not suitable during the subsequent preform formation due to their poor mechanical behavior, and the yarns are damaged by the preform process. Jiang et al. produced flax/PP hybrid yarns by the co-wrapping spinning method, in which PP filament was voluminously wrapped around flax fiber. The main spinning parameters were analyzed and optimized to obtain different flax ratios [8]. Corbin et al. developed hemp/PA12 hybrid yarn adapted for composite manufacture by using the co-wrapping method, and they characterized both the textile and tensile properties [14]. Bar et al. manufactured flax/PP hybrid yarns by using the DREF-3 spinning method for biocomposites. The effects of different process parameters and thermal treatments on hybrid yarn properties have been studied. It was observed that increasing the sheath ratio and the surface treatment temperature and decreasing the core twist level results in enhancement of further weavability of hybrid yarns [7]. Since the matrix form considered consisted of staple fibers, this method is not suitable for all material options and design choices.

Among these hybrid yarn techniques, axial micro-braiding and co-wrapping processes produce similar core/sheath hybrid yarn structure and possess similar advantages such as (1) the continuous reinforcing fibers, which maintain the untwisted form and straightness, exhibiting favorable mechanical properties that can be fully used and (2) the structure improving the coverage effect on reinforcing fibers, thus avoiding the damage during textile processing and reducing the hairiness. However, the key difference between the two techniques is that micro-braided yarn (MBY) consists of several helical interlacing wound tows, while there is no interlacement but just one tow voluminously wrapped in co-wrapped yarn (CWY) [15]. Does this difference influence the characteristics of the hybrid yarn? While there has been no comparative study of these methods yet, we compared them in this work by preparing hybrid yarns with the same core/sheath mass ratio, taking advantage of their similar core/sheath structures. At the same time, as we know, in each technique, the yarn parameters related to the resin also influence the performance. Hence, this article presents the results of a comparative study where we focused on resin parameters in hybrid yarns. Flax/PP MBY and CWY were developed with the same flax/PP mass ratio since they both have core/sheath structure, and then different PP parameters in each technique were varied. Textile and tensile properties in dry- and thermo-state were tested. MBYs’ and CWYs’ tensile properties were studied by comparing with those of the commingled yarn to determine the influence of hybrid techniques on reinforcing fibers; tensile properties of MBY and CWY with the same flax/PP mass ratio after the braiding process were compared with those before the braiding process to study thoroughly the effects of PP cover factors and different hybrid techniques on characteristics at the yarn scale. The two different hybrid yarn techniques were compared and evaluated in terms of yarn morphologies, structures, mechanical tensile properties, and braidabilities.

## 2. Materials and Methods

### 2.1. Raw Materials

Flax roving (Lincore flax untwisted roving, supplied by Groupe Depestele, Le Bocasse, France) was used as reinforcing fiber. Polypropylene (PP) multifilament (Polypropylene PPH 9069, supplied by TOTAL, Feluy, Belgium) was used as matrix fiber. The continuous filament fibers were prepared with the extrusion method. The main properties of raw materials are shown in Table 1.

### 2.2. Preparation of the Flax/PP Hybrid Yarns

**Figure 1:**
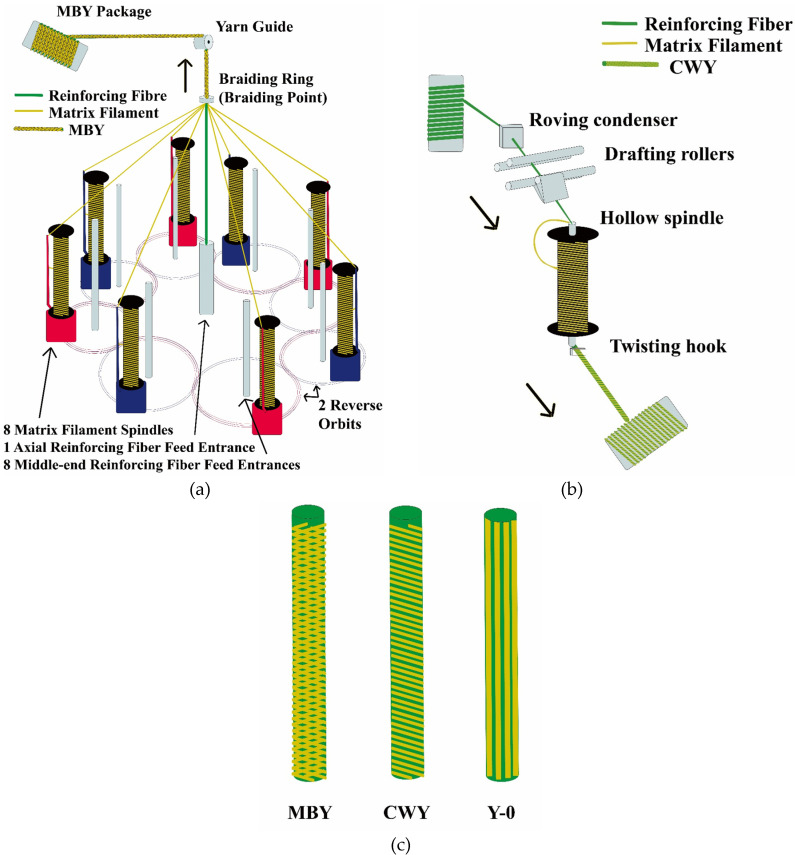
Schematic diagrams of manufacturing (**a**) micro-braided yarn (MBY) and (**b**) co-wrapped yarn (CWY). (**c**) Illustration of MBY, CWY and Y-0.

The micro-braided yarns (MBYs) were created by a tubular braiding loom with 8 spindles that hold bobbins with braider filaments. A schematic diagram of MBY manufacturing is shown in Figure 1a. Flax roving was used as the straightly and stably fed axial fiber; 8 bobbins with PP filaments moved along 2 reverse orbits, braiding around the reinforcing flax roving [17]. All the roving and filaments were ensured to be gathered at the braiding ring, where MBY was formed with a certain braiding angle [18]. By changing the ratio of PP braider bobbin speed to MBY take-up speed (i.e., the two parameters of the braiding loom), different PP braiding angles were obtained. PP braiding angles were measured by Image J software. Considering that the PP mass ratio should be between 35 and 65%, we selected 2 braiding angles: 20° (MBY-A) and 50° (MBY-B). 

The co-wrapped yarns (CWYs) were created by a hollow spindle spinning loom; the spinning process is illustrated in Figure 1b. Flax roving went through roving condenser and drafting rollers as a core roving then was guided into the hollow spindle; at the same time, PP filament wrapped around flax roving then went together in the hollow spindle. Flax roving and PP filament both had false twist since the hollow spindle rotated speedily. After passing through the twisting hook, the false twist of flax roving became untwisted, while PP filament wraps remained twisted [8]. The hollow spindle twist and hollow spindle rotational speed are two main parameters of the spinning process that determine the PP wrapping turns per meter. Considering that the PP mass ratio should be between 35 and 65%, we selected 2 PP wrapping turn numbers: 800 tpm (CWY-A) and 1000 tpm (CWY-B).

In addition, a flax/PP commingled yarn was made and named Y-0, as a reference yarn. It was assembled by straight and parallel mixing of the flax roving and 8 PP filaments, with no need for braiding or spinning looms. Its tensile properties were studied in comparison with MBYs and CWYs to determine the influence of hybrid techniques on reinforcing fibers (flax roving).

### 2.3. Textile Properties Testing

The textile properties of MBYs and CWYs were evaluated to understand exactly how the techniques and PP parameters influence the hybrid yarn characteristics. All the MBYs and CWYs were stored in a climatic chamber (T= 20 ± 3 °C, HR = 65 ± 3%) for at least 48 h to ensure that the materials reached moisture equilibrium. The linear density was measured according to NF G07-316 standard, with 10 cyclic tests. The uniformity and hairiness were measured by Uster Tester 4 machine. The uniformity is represented by the coefficient of variation of the weight or fiber number over the yarn length (CVm %). The hairiness index represents the total length of the protruding fibers concerning the sensing length of 1 cm of the yarn [19]; it was measured with 5 cyclic tests. The cover factor (CF) is used to measure the braiding filament deposition and is defined as the percent of the mandrel surface covered by the braiding tows [20]. Thus, the MBY and CWY cover factors (CF_MBY and CF_CWY) can be defined as the percentage of flax roving surface covered by PP filaments and are calculated by Equations (1) [21] and (2), respectively, where $W_{M}$ is PP filament width (0.60 mm), $N_{M}$ is number of PP filaments in MBY (here$\;N_{M}=8)$, $R_{F}$ is effective flax roving radius (0.47 mm), α is PP braiding angle in MBY, and $T_{M}$ is PP wrapping turns per meter in CWY.

(1) $$CF\_MBY=1-\left(1-\frac{W_{M}N_{M}}{4\pi R_{F}\cos\alpha }\right){\;}^{2}$$

(2) $$CF\_CWY=1-\left(1-\frac{W_{M}T_{M}}{1000}\right){\;}^{2}$$

### 2.4. Tensile Characterization

The dry-state tensile tests were performed on conditioned yarn specimens using a universal tensile machine MTS Criterion, according to NF EN ISO 2062 standard for a single yarn. The tensile test for each yarn was repeated 10 times to obtain an average value. The length of the specimen was 250 mm, preload was 0.5 ± 0.1 cN/tex, and the crosshead speed was set as 5 mm/min to obtain the accurate value since the deformation of flax roving is low. 

**Figure 2:**
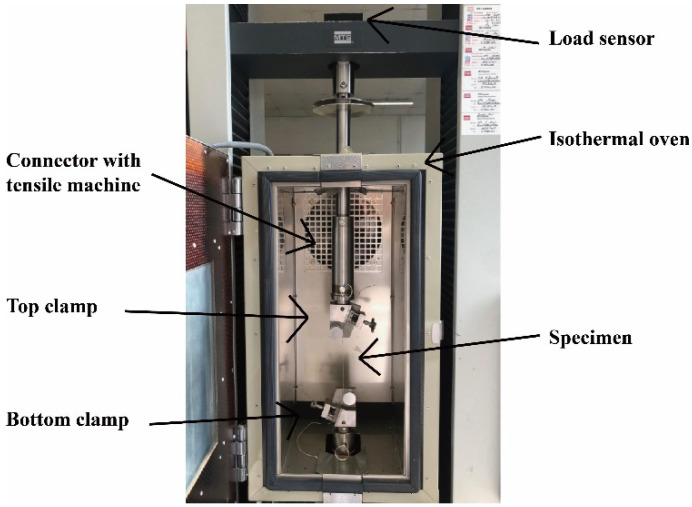
Thermo-state tensile test of single hybrid yarn.

The thermo-state tensile tests were conducted using a universal tensile tester MTS and an isothermal oven. Figure 2 shows the set-up of the machine. 

A single hybrid yarn was inserted and clamped by a top movable clamp. During the tests, a load sensor (10 kN) on the top measured the force in real time. The whole was process conducted in the oven, the length of the specimen was set at 150 mm considering the size of the oven, the temperature was set to 180 °C (greater than PP melting point (165 °C)), and the extension velocity was 5 mm/min. Different hybrid yarns were tested respectively to study the effects of the techniques on thermomechanical properties. The test temperature was reached by an augmentation phase at 20 °C/min. Once the test temperature was reached, the specimen temperature in the oven needed to be stabilized for 5 min before the extension [22,23]. Preload was set as 0.2 N, and there were 5 cyclic tests.

### 2.5. Braidability Testing

**Figure 3:**
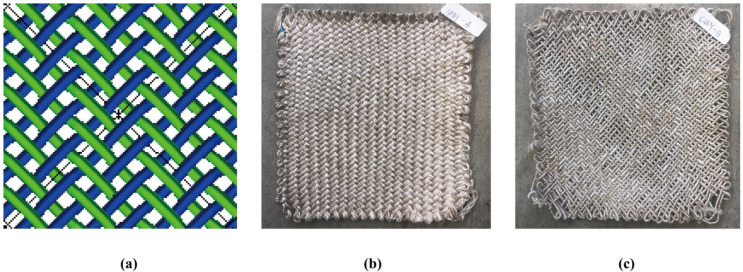
The 2D braiding: (**a**) illustration of braiding pattern (regular braid 2:2-1 overlap); (**b**) micro-braided yarn with 20° braiding angle (MBY-A) braids; (**c**) co-wrapped yarn with 1000 tpm (CWY-B) braids.

To study the yarn performance and braidability, 2D braids were conducted using MBY and CWY with the same size (200 × 200 mm^2^) and the same yarn density (2 yarns/cm), as shown in Figure 3. The preforms were conditioned at T = 20 ± 3 °C and HR = 65 ± 3% for at least 48 h. The yarns were carefully unbraided, and tensile testing was conducted to analyze the tensile characteristics before and after braiding preform. The specimens were chosen in different locations of the preforms; the length of the specimen was 250 mm, the preload was 0.5 ± 0.1 cN/tex, the crosshead speed was set as 5 mm/min, and there were 5 cyclic tests.

## 3. Results and Discussion

### 3.1. Textile Properties of the Flax/PP MBYs and CWYs

The morphological figures and measured textile properties of MBYs and CWYs are listed in Table 2; the difference between MBYs and CWYs can be found from the morphologies and data. It should be noted that Y-0 (linear density 1881 tex, PP mass ratio 43.9%), as a reference yarn, is not listed in this table. 

Flax rovings in all the MBYs and CWYs maintained the untwisted form and straightness, exhibiting favorable properties that can be fully used. With the same flax roving, the larger PP parameters (PP braiding angle for MBYs and PP wrapping turns for CWYs respectively) led to the larger linear densities of the hybrid yarns; i.e., the linear density of MBY-B was greater than that of MBY-A, and the linear density of CWY-B was greater than that of CWY-A. In MBYs, PP filaments were well and completely covered on flax roving for MBY-A (cover factor = 98.05%), while being over-covered for MBY-B (cover factor could not be calculated by Eq 1). When the PP braiding angle increased, the MBY-B became thinner and compact, having a higher PP mass ratio (increased to 49.5%), better uniformity (CVm = 10.12%), and less hairiness (3.11%) than MBY-A. Meanwhile, in the CWYs, PP filament coverage was homogeneous on flax roving for CWY-A (cover factor = 73%), and CWY-A had better uniformity (CVm = 14.86%) and less hairiness (3.80%). With the increase in PP wrapping turns to obtain a higher PP mass ratio, the coverage seemed inhomogeneous and the yarn was thicker for CWY-B, with overwrapping leading to PP bulking even though the cover factor was larger (84%). 

**Figure 4:**
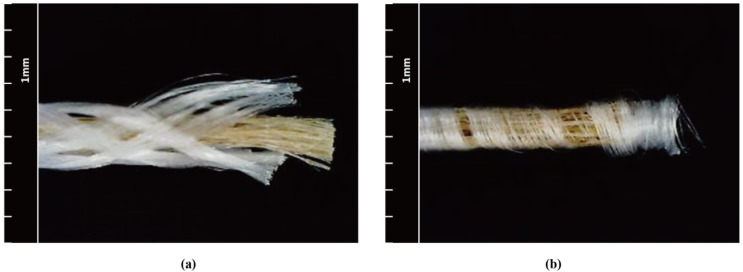
Structural comparison of (**a**) MBY-A vs. (**b**) CWY-B.

When comparing MBY-A and CWY-B, although they had similar flax/PP mass ratios (45.5% and 44.6%, respectively), MBY-A presented a better morphology, as it is easier to obtain a higher PP mass ratio with the technique micro-braiding than the co-wrapping technique. The structures of MBY-A and CWY-B are shown in Figure 4. CWY-B (width less than 2 mm) was thinner than MBY-A (width greater than 2 mm) since the PP filament was wrapped voluminously. MBY-A, which presented better uniformity (CVm = 11.87%) and less hairiness (3.48%), was softer than CWY-B (16.79% CVm and 5.37% hairiness). What is more, a light touch of hand would lead to movement of the wrapped PP on CWY-B, causing it to become inhomogeneous; thus, the yarn manufactured by the co-wrapping technique always had structural instability. 

### 3.2. Tensile Results 

#### 3.2.1. Dry-State Tensile Properties

**Figure 5:**
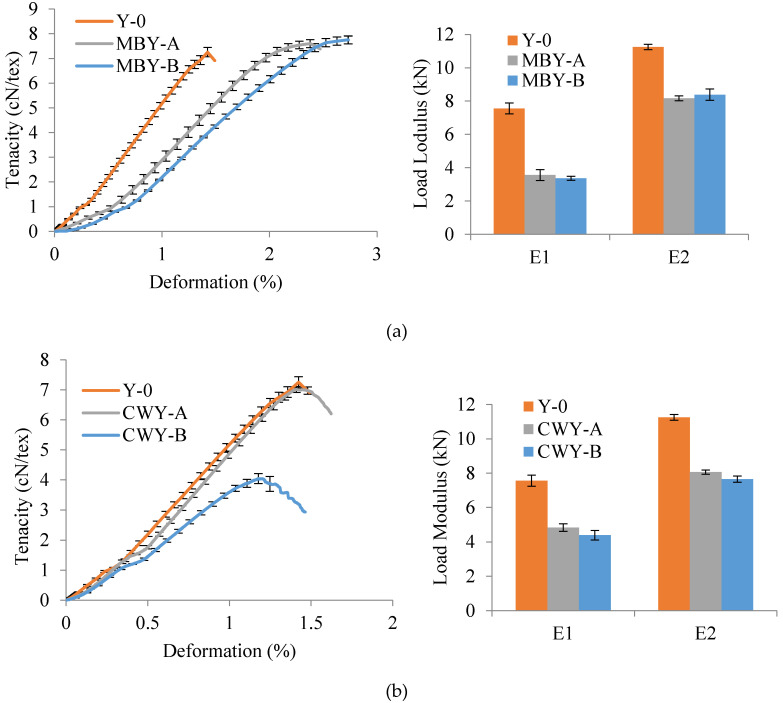

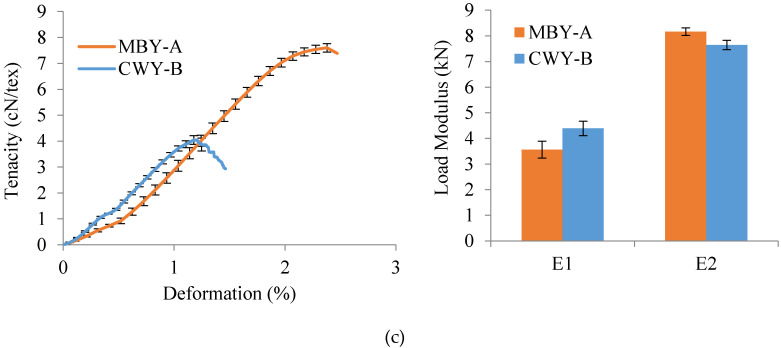
Dry-state tensile results of (**a**) MBYs vs. Y-0, (**b**) CWYs vs. Y-0 and (**c**) MBY-A vs. CWY-B.

For textile flax hybrid yarns, the mechanical properties cannot be calculated as force per unit area of cross-section as it is very difficult to measure the cross-section for each yarn with natural flax. Consequently, load modulus (kN) was used to quantify the tensile results. The tensile load modulus is calculated as the slope of the initial force vs. deformation curve. From Table 1, we know that the flax roving had high strength but very short deformation, so the breakage of MBY and CWY was firstly the breakage of core flax roving. The fracture of flax roving surface treatment agent divided the curve into two deformation phases: E1 and E2. For MBY, E1 was between 0 and 0.5% and E2 was between 1.4 and 1.8%, while for Y-0 and CWY E1 was between 0 and 0.2% and E2 was between 0.5 and 1% since the deformation of pure flax is extremely short. The representative results of dry-state tensile tests are shown in Table 3 and Figure 5.

In Y-0, i.e., the reference, there was no damage and no cohesive effect between PP filaments and flax roving, so the tensile test results matched the pure flax roving tensile properties shown in Table 1. In MBYs, as the PP braiding angle appeared and increased, MBY-A and MBY-B exhibited voluminous arrangement and better cohesive effect, and thus the Fmax, deformation at Fmax, and tenacity were greater than those of Y-0 (9.9% increase for MBY-A and 12.0% increase for MBY-B). The larger PP braiding angle led to the larger coverage of flax and the greater frictional damage to the surface treatment agent. Y-0 had no frictional damage, so the E1 of Y-0 was the largest (7.56 ± 0.33 kN); E1 decreased both when PP braiding angle appeared (3.56 ± 0.33 kN for MBY-A) and increased (3.37 ± 0.12 kN for MBY-B). As for E2, Y-0′s was the largest (11.25 ± 0.17 kN), and E2 decreased (8.17 ± 0.14 kN for MBY-A) when the PP braiding angle appeared; since there was no surface treatment agent in phase E2, as the PP braiding angle increased, the E2 became larger (8.39 ± 0.34 kN for MBY-B) because of the better PP cohesive effect. In CWYs, the technique has the process of false twist and untwist on flax roving, which itself damages flax roving. Therefore, when the PP wrapping appeared, CWY-A had a smaller Fmax, smaller tenacity, and smaller E1 and E2 than Y-0 (7.1% decrease); when the PP wrapping turns increased, the damage to flax roving became greater, and therefore CWY-B presented worse behavior (47.8% decrease) than Y-0. 

Comparing MBY-A and CWY-B, it can be seen that MBY-A had larger Fmax and tenacity than CWY-B. It can be noted that the micro-braiding technique provided a good cohesive effect between PP filaments and flax; on the contrary, the co-wrapping technique itself damaged the reinforcing fibers. That is why even the E1 of MBY-A (3.56 ± 0.33 kN) was less than that of CWY-B (4.39 ± 0.28 kN), but in the E2 phase, thanks to the cohesive effect, E2 of MBY-A (8.17 ± 0.14 kN) was larger than that of CWY-B (7.65 ± 0.18 kN). Therefore, with the same core/sheath mass ratio, MBY had better dry-state tensile behavior than CWY.

#### 3.2.2. Thermo-State Tensile Properties

**Figure 6:**
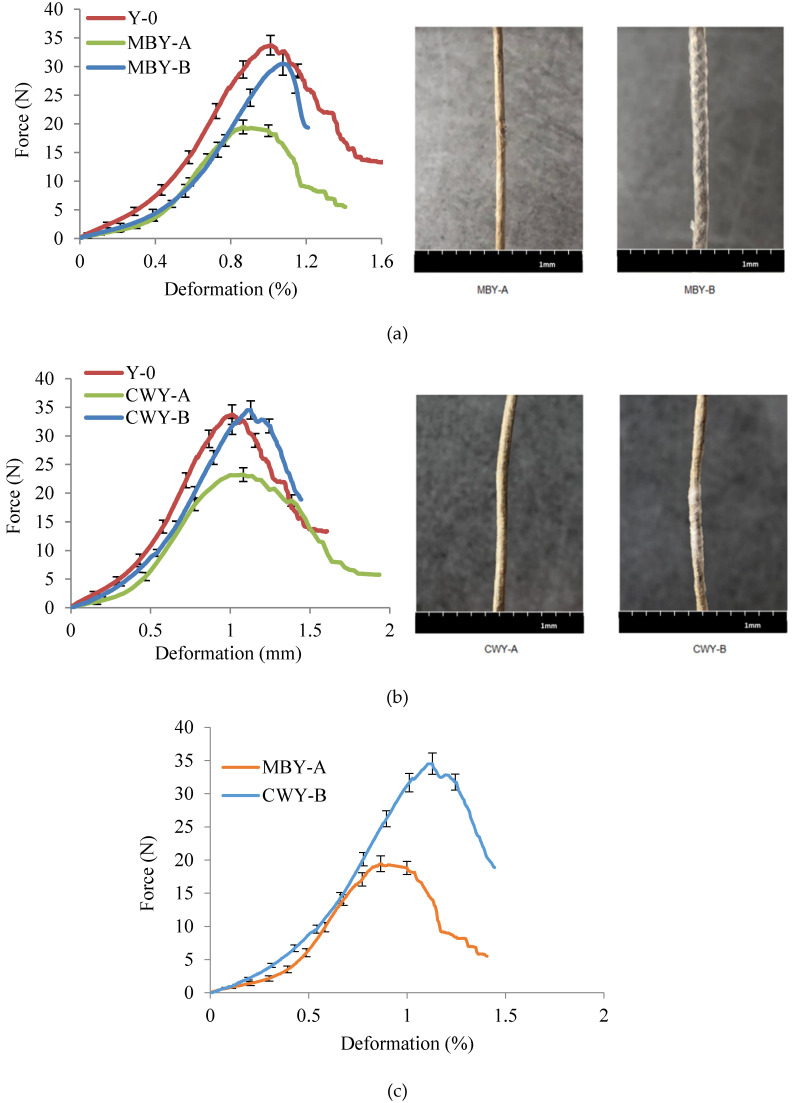
Thermo-state tensile results of (**a**) MBYs vs. Y-0, (**b**) CWYs vs. Y-0, and (**c**) MBY-A vs. CWY-B.

The forces applied versus the deformation of MBYs and CWYs are presented in Figure 6. Comparing with Y-0 (Fmax 33.7 N), when the PP parameters appeared, the Fmax declined obviously. MBY-A (Fmax 19.5 N) and CWY-A (Fmax 23.2 N) had the same trend, and this was related to the PP sheath structure being well covered and creating more lubrication effect in flax roving. In contrast, when the PP parameters increased, the Fmax of MBY-B (30.5 N) was greater than that of MBY-A and the Fmax of CWY-B (34.5 N) was greater than that of CWY-A. By examining the morphology after heating, it can be easily seen that for the MBY-B, the excessive arrangement of PP with 50° braiding angle led to the low utilization rate of PP and worse impregnation. The MBY-B was particularly hard in comparison to MBY-A. A similar situation was observed for CWY-B: even though the PP mass ratio of CWY-B was larger than that of CWY-A, the bulking, inhomogeneous PP and incomplete coverage led to the low utilization rate of PP and worse impregnation. 

Comparing MBY-A and CWY-B, from the morphologies after heating in Figure 6, we can see that the MBY-A had a better impregnation than CWY-B, while CWY-B even presented some dry areas where there was no impregnation. Therefore, the lubrication effect in flax roving for MBY-A was better, the Fmax of MBY-A (19.5 N) was smaller than that of CWY-B (34.5 N), and the deformation at break was smaller as well. This means that even with same mass ratio, different techniques result in different thermo-behaviors. MBY-A presented better morphology in the dry-state and better impregnation during the thermo-state, but CWY-B presented bulking, inhomogeneous PP and incomplete coverage in the dry-state, which led to a low utilization rate of PP and worse impregnation during thermo-state. 

### 3.3. Braidability of the MBY and CWY

**Figure 7:**
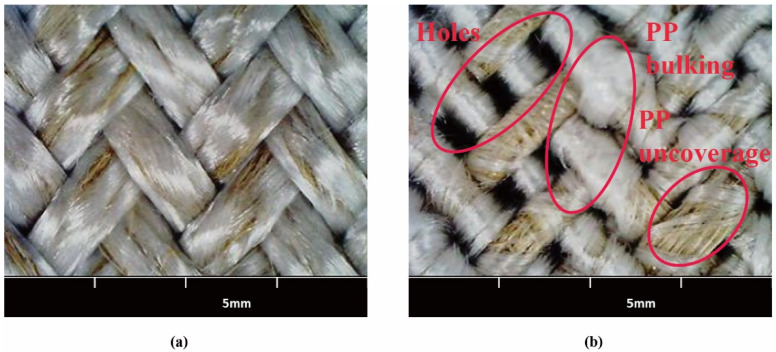
The morphologies of the same yarn density braiding preforms: (**a**) MBY-A braids and (**b**) CWY-B braids.

The morphologies of the same yarn density braiding preforms with the same flax/PP mass ratio hybrid yarns (MBY-A and CWY-B) are shown in Figure 7. Since the wrapped PP of CWY-B was easy to move during the braiding process, there was PP bulking and insufficient coverage. Meanwhile, the CWY-B yarn was relatively thin and very hard and was not flexible for the preform process, which led to the CWY-B preform having many holes with the same yarn density as MBY-A preform. On the contrary, the MBY-A preform was very neat and flat, the PP filaments covered and protected the flax roving during the braiding process. 

**Figure 8:**
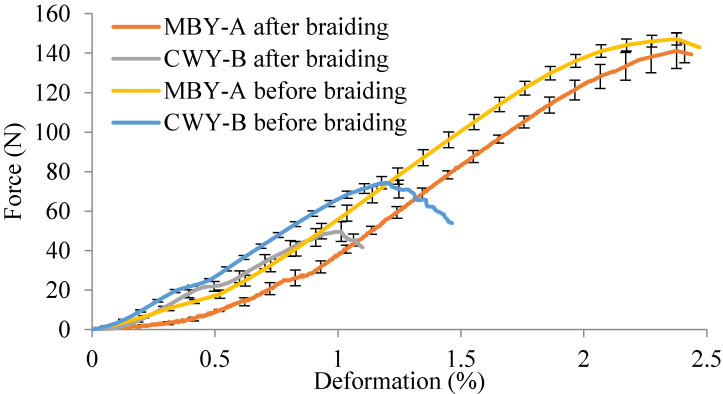
Force vs. deformation curves of MBY-A and CWY-B before and after braiding.

The tensile tests were conducted after the braiding process on yarns extracted from the braids. To demonstrate the hybrid yarns’ braidabilities and the influence of the braiding process, it is interesting to report the tensile behaviors of MBY-A and CWY-B before and after braiding. The influence of the braiding process at the yarn scale can be observed in Figure 8. A smooth curve in the beginning phase can be both noted for MBY-A and CWY-B; this is because that the braiding process caused the shrinkage of the yarn. The Fmax did not change much for MBY-A before and after braiding, taking into account the standard deviations. Meanwhile, the Fmax of CWY-B after braiding (49.6 N) decreased observably compared to that before braiding (74.4 N). Since the braiding process has an impact on the yarns, assuming that the linear densities of hybrid yarns before and after the braiding process remain the same, the tenacities were calculated to evaluate the influence. The comparison of tenacities before and after braiding was calculated and the results are reported in Table 4, with the percentage decrease also being presented in this table. Regarding the tenacity, there was a decrease of up to 33.0% for CWY-B after braiding, while there was only a 4.3% decrease for MBY-A. 

The comparison of the load modulus before and after braiding was also calculated, and the results are reported in Table 5, with the percentage decrease of yarn modulus after braiding also being presented. Compared to the load modulus before braiding, the load modulus after braiding decreased for both hybrid yarns. Consequently, it can be said that the braiding process generally led to a reduction of the yarn stiffness. A significant drop in the first load modulus after the braiding process was noted for both the yarns (up to 56% for MBY-A), which was because of the yarn shrinkage. It can be noted that the braiding process had a higher impact on the stiffness on CWY-B than MBY-A (20% for CWY-B and 7% for MBY-A), which was because the uneven coverage exposed part of the flax roving and the braiding process damaged the flax, thereby reducing the stiffness of the yarn. After the braiding process, the MBY remained quasi-equivalent to E2 before the braiding process; the well-covered PP filaments on MBY had a positive influence in preventing the reduction of the yarn stiffness during the braiding. 

## 4. Conclusions

This study has presented a comparison of the micro-braiding technique and co-wrapping technique with the same flax/PP mass ratio and a comparison of different PP parameters in each technique. The effects of PP braiding angles in MBYs, PP wrapping turns in CWYs, and different techniques on textile and mechanical characteristics and yarn braidability have been discussed. Larger PP parameters, namely PP braiding angle for MBY and PP wrapping turns for CWY, normally result in better textile and tensile performance of hybrid yarn, but extremely large PP parameters will lead to over-coverage and a lower PP utilization rate, thus causing a pore impregnation effect. 

Compared to the co-wrapping technique, the micro-braiding technique presents several advantages: (1) The micro-braiding technique can more easily obtain the desired core/sheath ratio, while the co-wrapping technique simply uses one single filament to voluminously wrap, making it difficult to achieve a high PP mass ratio. (2) The micro-braiding technique has superior structural stability. With the helical interlacing wound tow structure, the morphology of MBY is uniform and smooth, whereas the CWY obtained is inhomogeneous with easily moveable PP filament indicative of unstable structure. (3) The better mechanical properties of the micro-braiding technique represent the most important advantage. With its unique braiding structure, the micro-braiding technique provides a synergistic cohesive effect between PP filaments and flax roving, which can increase the total stiffness of the yarn by 12.0%. Meanwhile, with the high hollow spindle rotation speed as well as the false twisting and untwisting process, the co-wrapping technique causes great damage to flax roving, leading to a 47.9% decrease in stiffness. (4) With better textile and tensile properties, MBY presents a better braidability than CWY because of the good protection of flax roving. In comparison, CWY is less suitable for the braiding process because it is hard and not flexible. The co-wrapping technique is commercially viable, while the production rate of the micro-braiding technique is relatively slow. Overall, both techniques open up a broad prospect for the development of thermoplastic biocomposites. 

## Figures and Tables

**Table 1:** The main properties of raw materials.

	Density (g/cm^3^)	Linear Density (Tex)	Thermodynamic Temperature (C°)	Tenacity (cN/tex)	Deformation (%)
Flax Roving	1.45 [16]	1000	325–360 ^1^	13.64 ± 0.42	1.42 ± 0.03
PP Filament	0.91	97.50	165 ^2^	18.98 ± 0.23	30.25 ± 0.05

^1^ Decomposition temperature (thermogravimetric analysis). ^2^ Melting temperature.

**Table 2:** Morphologies and textile properties of MBYs and CWYs.

	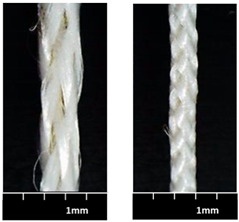		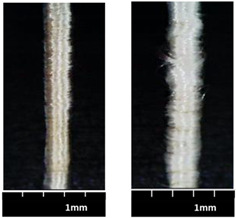	
Hybrid yarn ID	MBY-A	MBY-B	CWY-A	CWY-B
PP Parameter	PP Braiding Angle (°)		PP Wrapping Turns (tpm)	
	20	50	800	1000
Linear Density (tex)	1936 ± 27	2219 ± 17	1450 ± 14	1838 ± 28
PP Mass Ratio (%)	45.5	49.5	32.0	44.6
CVm (%)	11.87	10.12	14.86	16.79
Hairiness (H)	9.35	8.05	11.74	13.25
sH (%)	3.48	3.11	3.80	5.37
Cover Factor (%)	98.05	N/A	73	84

**Table 3:** Dry-state tensile properties of MBYs and CWYs.

Hybrid Yarn ID	Force-Max (N)	Deformation at Fmax (%)	Tenacity (cN/tex)	Load Modulus E1 (kN)	Load Modulus E2 (kN)
Y-0	136.46 ± 3.53	1.42 ± 0.03	7.25 ± 0.19	7.56 ± 0.33	11.25 ± 0.17
MBY-A	147.15 ± 3.11	2.38 ± 0.05	7.60 ± 0.16	3.56 ± 0.33	8.17 ± 0.14
MBY-B	172.04 ± 3.54	2.73 ± 0.12	7.75 ± 0.16	3.37 ± 0.12	8.39 ± 0.34
CWY-A	101.68 ± 1.47	1.41 ± 0.03	7.01 ± 0.10	4.83 ± 0.21	8.06 ± 0.13
CWY-B	74.36 ± 3.09	1.18 ± 0.07	4.05 ± 0.17	4.39 ± 0.28	7.65 ± 0.18

**Table 4:** Hybrid yarn tenacities before and after the braiding process.

Hybrid Yarn ID	Tenacity before Braiding (cN/tex)	Tenacity after Braiding (cN/tex)	Percentage Decrease of Yarn Tenacity after Braiding (%)
MBY-A	7.60 ± 0.16	7.27 ± 0.31	4.34
CWY-B	4.05 ± 0.17	2.70 ± 0.24	33

**Table 5:** Hybrid yarn load modulus (E1 and E2) before and after braiding.

Hybrid Yarn ID	Load Modulus before Braiding (kN)		Load Modulus after Braiding (kN)		Percentage Decrease of Yarn Modulus after Braiding	
	E1	E2	E1	E2	E1 (%)	E2 (%)
MBY-A	3.56 ± 0.33	8.17 ± 0.14	1.57 ± 0.31	7.59 ± 0.11	56	7
CWY-B	4.39 ± 0.28	7.65 ± 0.18	2.56 ± 0.24	6.13 ± 0.14	42	20
